# Liver Injury Indicating Fatty Liver but Not Serologic NASH Marker Improves under Metformin Treatment in Polycystic Ovary Syndrome

**DOI:** 10.1155/2015/254169

**Published:** 2015-03-19

**Authors:** Susanne Tan, Nils Vollmar, Sven Benson, Jan-Peter Sowa, Lars P. Bechmann, Guido Gerken, Dagmar Fuhrer, Ali Canbay

**Affiliations:** ^1^Department of Endocrinology and Division of Laboratory Research, University Hospital, University of Duisburg-Essen, Hufelandstraße 55, 45147 Essen, Germany; ^2^Institute of Medical Psychology and Behavioral Immunobiology, University Hospital, University of Duisburg-Essen, Hufelandstraße 55, 45147 Essen, Germany; ^3^Department of Gastroenterology and Hepatology, University Hospital, University of Duisburg-Essen, Hufelandstraße 55, 45147 Essen, Germany

## Abstract

*Objective*. Polycystic ovary syndrome (PCOS) is associated with obesity and insulin resistance (IR), key features of nonalcoholic steatohepatitis (NASH). Cytokeratin 18 fragments (M30) have been established as a serum marker for NASH. The insulin sensitizer metformin improves hepatic IR. This study evaluates the influence of MF on serologic NASH (sNASH) in patients with PCOS.* Patients and Methods*. In 89 patients, metabolic parameters, liver injury indicating fatty liver (LIFL), and M30 were assessed at baseline and after metformin treatment. Patients with initial IR were subdivided into dissolved (PCOS-exIR) and persistent IR (PCOS-PIR) after treatment and compared to an initially insulin sensitive PCOS group (PCOS-C).* Results*. Improvement of LIFL prevalence could be seen in PCOS-C and PCOS-exIR compared to PCOS-PIR (−19.4, resp., −12.0% versus 7.2%, Chi^2^ = 29.5, *P* < 0.001) without change in sNASH prevalence. In PCOS-PIR, ALT levels increased significantly accompanied by a nominal, nonsignificant M30 increase.* Conclusions*. Metformin improves LIFL in subgroups of patients with PCOS without influencing sNASH. This could either indicate a missing effect of metformin on NAFLD or slowed disease progression. Further studies are needed to elucidate NAFLD in the context of PCOS and potential therapeutic options.

## 1. Introduction

The polycystic ovary syndrome (PCOS) is a common endocrinopathy affecting at least 5–10% of women in childbearing years [1]. It is classically characterized by hyperandrogenism, chronic anovulation, and polycystic ovarian morphology in ultrasonography [2]. Although the underlying pathophysiological mechanisms of PCOS remain unclear, insulin resistance (IR) intrinsic to the syndrome appears to play a central role in its development. Presence of IR in PCOS is partially explained by obesity, but it is also found in lean women with PCOS [3]. Given the significant metabolic burden of IR seen in women with PCOS, affected women may have an increased risk of type 2 diabetes mellitus, cardiovascular disease [4, 5], and also nonalcoholic fatty liver disease (NAFLD) [6–11].

NAFLD is the most common form of liver disease, with a prevalence of 5–33% in the general population. Simple steatosis hepatis (SH), the most common form of NAFLD, typically follows a benign clinical course. However, the progressive form, nonalcoholic steatohepatitis (NASH), is a potentially serious condition resulting in progression to cirrhosis in 25% of these patients, including the long-term complications of portal hypertension, liver failure, and hepatocellular carcinoma [12, 13]. The latter may also occur in NASH without evidence of cirrhosis [14]. As in PCOS, IR appears to play a key role in NAFLD development [15–17]. Diabetes has also been described as a risk factor for SH progression to fibrosis [18]. Although still serving as the gold standard for differentiating between SH and NASH, liver biopsy can result in various complications due to its invasive character. Alternatively, apoptotic cell death may serve as a noninvasive method to evaluate NASH. Hepatocyte apoptosis plays an important role in liver injury and disease progression in NASH and various other liver diseases [19–23]. Indeed, hepatocyte cell death by apoptosis is typically enhanced in NASH but absent in SH [24]. In fact, Feldstein and colleagues [25, 26] have reported that cell death index can replace liver biopsy to diagnose NASH in a cohort of patients with various types of liver disease. Induction of apoptosis by such events as accumulation of free fatty acids in the liver cells activates effector caspases that cleave a host of intracellular substrates including cytokeratin 18 (CK18), a member of the intermediate filament family of cytoskeletal proteins [27]. Caspase-3-dependent cleavage of CK18 at Asp396 exposes a neoepitope, M30, which reflects hepatic apoptosis [28]. Recently, it has been shown that the plasma-borne caspase-generated CK18 fragments independently predict NASH in multivariate analysis [25, 26]. Although not assessed in routine clinical practice, the American College of Gastroenterology and the American Gastroenterological Association regard CK18 as a promising biomarker for identifying steatohepatitis [29].

PCOS is highly associated with NAFLD diagnosed by aspartate aminotransferase (AST) elevation and/or ultrasound [6–8]. Furthermore, in a small number of patients with PCOS and persistently elevated liver enzymes, liver biopsy revealed NASH with advanced fibrosis [10]. Conversely, 10 of 14 (71%) female patients in childbearing years with histologically diagnosed NAFLD also had revealed PCOS [9], indicating a close relationship between these two entities. Using CK18 fragments as a surrogate parameter of apoptotic cell death, we could demonstrate a high risk for NASH in PCOS with up to a quarter of patients with PCOS fulfilling the criteria for serologic NASH previously. Further analysis revealed a small but significant effect of IR on NASH markers [11].

As IR plays a key role in the pathophysiology of PCOS, insulin sensitizing agents are widely studied and used in the treatment of PCOS, with metformin (MF) as the most commonly used pharmacological agent. MF acts as an insulin sensitizing agent resulting in a reduction of hepatic glucose secretion and increase of peripheral glucose utilization with positive effects on IR, body weight, and menstrual cycling in PCOS [30]. Improving insulin sensitivity by MF may positively influence the development of NASH, for example, by suppressing acetyl-CoA carboxylase activity and accumulation of adenosine monophosphate in hepatocytes resulting in inhibition of mitochondrial fatty acid oxidation and blocking glucagon-dependent glucose output from hepatocytes [31, 32]. Some studies report a beneficial effect of MF on liver function with improvement of liver enzymes or even improvement of tissue steatosis or inflammation in NAFLD and/or NASH [33–38]. Though there is also conflicting data [39–42], two studies demonstrated improvement of liver enzymes in patients with PCOS under MF treatment, indicating that MF positively influences liver function [43, 44].

PCOS is a frequent endocrinopathy and includes a high percentage of young women, who are potentially at high risk for serious liver disease, and IR plays a key role in the pathophysiology of both entities, PCOS and NAFLD. We hypothesize that a six-month therapy with MF positively influences serologic defined NASH by improving IR in patients with PCOS.

## 2. Patients and Methods

### 2.1. Participants

Consecutive patients with newly diagnosed, currently untreated PCOS with and without IR (*n* = 89) were retrospectively recruited at the Outpatient Clinic of the Department of Endocrinology and Division of Laboratory Research, University of Duisburg-Essen, Germany. PCOS was defined according to the Rotterdam criteria; therefore, diagnosis of PCOS was established if two or more of the criteria, hyperandrogenism, chronic anovulation, or polycystic ovaries, were fulfilled and other pituitary, adrenal, or ovarian diseases could be excluded [2].

### 2.2. Clinical Characterization

Participants were carefully characterized with regard to medical history and clinical and sociodemographic variables using questionnaires, interview, and physical examination, as previously described in detail [30]. Free androgen index (FAI) was calculated as total testosterone (nmol/liter) ∗ 100/SHBG (nmol/liter). IR was defined as elevation of homeostasis model assessment of IR (HOMA-IR > 2.5) [45–47]. HOMA-IR > 2.5 was also suggested as cut-off to discriminate between patients with biopsy proven NAFLD or patients with NAFLD diagnosed by ultrasound and elevated liver enzymes with a specificity of 94% and a sensitivity of 74% [48]. Metabolic syndrome (MBS) was defined according to NCEP/ATP guidelines when 3 of the 5 following criteria were fulfilled: (1) waist circumference > 88 cm, (2) triglycerides ≥ 150 mg/dL, (3) HDL-cholesterol < 50 mg/dL, (4) blood pressure ≥ 130/85 mmHg, and (5) fasting glucose ≥ 110 mg/dL. Liver injury implicating fatty liver (LIFL) has been defined as elevation of aspartate aminotransferase (AST) or alanine aminotransferase (ALT) above the upper normal range (AST or ALT > 30 U/l) in the absence of relevant alcohol consumption or known chronic liver disease. BARD-Score (BMI, AST/ALT-ratio, diabetes mellitus Score) was calculated to evaluate risk for advanced fibrosis, [49]. As diabetes mellitus represented an exclusion criterion, possible reached maximum points in BARD-Score were 3 (if BMI ≥ 28 kg/m²: 1 point and if AST/ALT-ratio ≥ 0.8: 2 points; presence of diabetes mellitus: 1 point and as no patient had diabetes: always 0 points). M30 was used as serum surrogate parameter of NASH and levels ≥ 395 U/liter were defined as serologic defined NASH (sNASH) [26]. Any known or newly detected diabetes mellitus represented an exclusion criterion. Alcohol consumption greater than 20 g/d and other previously known or newly detected secondary reasons of liver diseases such as viral hepatitis, hemochromatosis, Wilson's disease, autoimmune diseases, and hepatotoxic drugs represented an exclusion criterion [50].

### 2.3. Study Design

**Figure 1:**
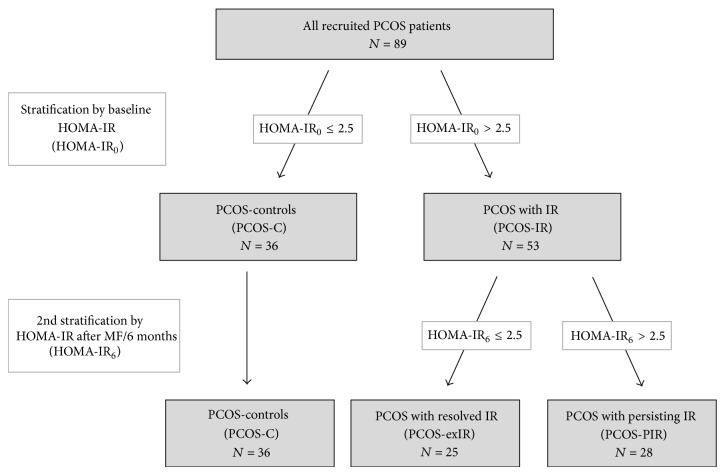
Stratification of PCOS patients according to HOMA-IR.

We performed a retrospective observational intervention study. Patients were evaluated at baseline and following treatment with MF in a weight-adapted dose for six months (body weight < 60 kg: 1000 mg, 60–100 kg: 1700 mg, and >100 kg or BMI ≥ 30 kg/m²: 2000 mg daily). They were divided into two groups according to presence or absence of IR defined by HOMA-IR > 2.5. Fifty-three patients with IR (PCOS-IR) were compared to a control group of 36 patients without IR (PCOS-C). According to therapy success defined by HOMA-IR normalization after metformin treatment, the PCOS-IR group was subdivided into a group with persistent IR (PCOS-PIR) and a group with dissolved IR (PCOS-exIR) (see Figure 1). The primary outcome of the study included the prevalence of sNASH and LIFL. Secondary outcome parameters included testosterone levels, BMI, parameters of IR, lipid status, liver enzymes, and apoptotic marker M30 as well as prevalence of MBS. The study protocol was approved by the Ethics Committee of the University of Essen. All subjects gave written informed consent before entering the study.

### 2.4. Biochemical Analyses

Automated chemiluminescence immunoassay systems were used for the determination of LH, FSH, TSH, testosterone, estradiol, cortisol, free T4, prolactin, blood glucose, AST, ALT (ADVIA Centaur; Siemens, Eschborn, Germany), ACTH, dehydroepiandrosterone sulfate, androstenedione, SHBG, insulin, and IGF (Immulite 2000, Siemens). Measurement of blood glucose was performed by photometric determination (ADVIA 2400, Siemens). Intra- and interassay variation were less than 5%, respectively, and 8% for all measured variables. 17-Hydroxyprogesterone was measured by the Biosource 17-OH-RIA-CT kit (Biosource International, Camarillo, CA) provided by IBL Hamburg (IBL, Gesellschaft für Immunchemie und Immunbiologie, Hamburg, Germany). The intra- and interassay coefficients of variation were 5.6 and 7.2%. Sera were collected upon admission and stored within 2 h at −20°C until testing. CK18 fragments were assessed by monoclonal antibody M30 using the M30-Apoptosense ELISA kit (Peviva, Bromma, Sweden) as previously described [51].

### 2.5. Statistical Analyses

1. Patients who were insulin sensitive at baseline (PCOS-C) and patients with IR at baseline were compared using independent samples *t*-tests or Chi²-tests.
2. For all subsequent analyses, patients with IR were subdivided into patients with insulin resistance after treatment (PCOS-PIR) and initially insulin resistant patients whose IR dissolved after treatment (PCOS-exIR). To evaluate treatment effects, repeated measures analyses of variance (ANOVA) were computed with the main factors group (PCOS-C, PCOS-PIR, and PCOS-exIR) and time (baseline, after 6-month MF treatment). In case of significant ANOVA treatment (time) or treatment × group interaction effects, post hoc comparisons of means with Bonferroni corrections were calculated using paired *t*-tests within PCOS groups. For nonparametric parameters (i.e., BARD-Score), Wilcoxon tests were computed.
3. To additionally explore differences in treatment effects between PCOS groups, delta scores (i.e., percent changes from baseline to six-month MF treatment) were computed for dichotomous variables (MBS, LIFL, and sNASH prevalence), and patient groups were compared using Chi²-tests.
4. To exclude that treatment effects (or differences in treatment effects between PCOS groups) were attributable to body weight reduction, all ANOVA analyses were repeated with delta body weight as covariate. Since body weight reduction did not affect any result, these data are not presented.

All results are shown as mean ± standard deviation unless otherwise indicated. The alpha level was set at 0.05. All data were analyzed with PASW 21.

## 3. Results

### 3.1. Pretreatment Characteristics

The majority of patients presented with IR at the beginning of MF therapy (53/89, 59.6%). Characteristics of patients are presented in Table 1. By definition, patients with IR had significantly higher parameters of IR. Testosterone concentration was similar in both groups, but FAI levels were significantly higher in insulin resistant patients. Furthermore, patients with IR presented with an adverse metabolic profile with significantly higher BMI, body weight, and triglycerides and lower HDL and therefore fulfilled 12-fold more often the criteria for MBS. AST/ALT-ratio was lower in patients with IR. Liver enzymes, BARD-Score, and M30 as well as prevalence of LIFL and sNASH were similar in both groups.

### 3.2. Effects of Metformin Intervention on IR

Regarding the whole cohort, prevalence of IR significantly declined after six-month metformin therapy (59.6% versus 33.7%, *P* < 0.001). In detail, normalization of IR was achieved in 47.2% (25/53, PCOS-exIR) of cases, while two of 36 patients developed IR during the treatment period (5.6%) and 38.2% patients remained insulin sensitive (34/89) and 31.5% remained insulin resistant (28/89, PCOS-PIR), respectively. PCOS-exIR and PCOS-PIR showed significantly greater improvements in HOMA-IR (*F* = 8.5, *P* < 0.001, interaction effect) and fasting insulin (*F* = 9.0, *P* < 0.001, interaction effect) than the PCOS-C group. Furthermore, PCOS-exIR patients showed an improvement of AUCI (for post hoc comparisons, see Table 2).

### 3.3. Effects of Metformin Intervention on Metabolic Parameters

**Figure 2:**
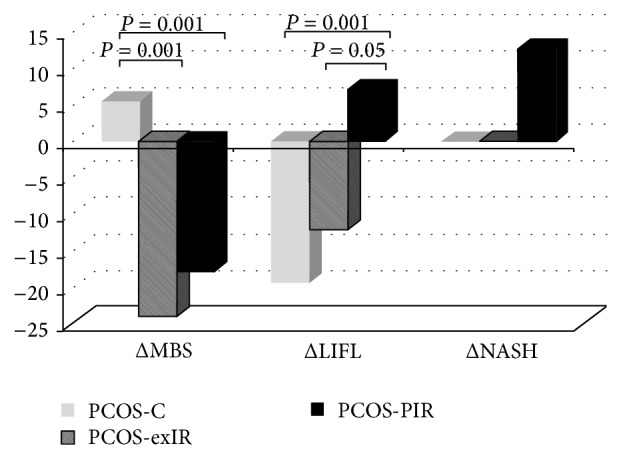
Comparison of metabolic/hepatic markers in the three PCOS groups (PCOS-C: patients who were insulin sensitive at baseline; PCOS-PIR: patients with IR at baseline and after treatment; PCOS-exIR: initially insulin resistant patients whose IR dissolved after treatment). Data are presented as percent changes (between baseline and after treatment) of prevalence and were analyzed using Chi²-tests.

A significant loss in body weight (−6 kg) and BMI (−2.2 kg/m²) was observed only in PCOS-exIR patients (body weight: *F* = 6.3, *P* < 0.01; BMI: *F* = 6.3, *P* < 0.01, interaction effects; for post hoc comparisons, see Table 2). MF treatment led to a slight but significant increase in HDL-cholesterol (*F* = 4.5, *P* < 0.05), while no significant changes in cholesterol, LDL-cholesterol and triglycerides could be demonstrated in all patients (Table 2). Both PCOS-exIR and PCOS-PIR groups showed significantly greater reductions in MBS prevalence (delta MBS) compared to PCOS-C (Chi² = 43.0, *P* < 0.001; Figure 2).

### 3.4. Effects of Metformin Intervention on Liver Function

Whereas PCOS-C and PCOS-exIR groups showed improvement of liver enzymes (i.e., a significant reduction of ALT), ALT concentration increased in PCOS-PIR patients (*F* = 7.6, *P* < 0.001, interaction effect; Table 2). In comparison to PCOS-PIR patients, decreases in LIFL prevalence were significantly greater in PCOS-C and PCOS-exIR patients (Chi² = 29.5, *P* < 0.001; Figure 2). AST/ALT-ratio, BARD-Score, M30 levels, and sNASH prevalence did not change significantly under treatment. Patients with PIR showed a nominal increase in levels of liver enzymes and LIFL prevalence and in M30 levels and sNASH prevalence. However, the magnitude of changes in these parameters (i.e., delta values) did not differ significantly between PCOS groups (Figure 2). To exclude an effect of MF-induced body weight reduction on any other outcome parameter (excluding BMI), ANOVA was additionally performed with change of body weight as covariate. All effects reported herein remained statistically significant (data not shown).

## 4. Discussion

In the presented study, we describe the effect of MF not only on liver enzymes but also on hepatic apoptotic markers as serologic parameter for NASH in PCOS. This study demonstrates that MF has a positive effect on liver enzymes in patients with PCOS, who were insulin sensitive, and in those reaching insulin sensitivity under MF. This effect was independent of achieved weight loss. However, even in the presence of treatment response for IR, this was not accompanied by improvement of apoptotic markers or sNASH prevalence. In contrast, patients with PCOS who did not demonstrate improvement in insulin sensitivity with MF treatment demonstrated worsening of liver enzymes with a nominal increase in apoptotic cell death marker M30.

A positive influence on liver enzymes by MF in PCOS has been described by two study groups. In an Italian study cohort, not only a high prevalence of NAFLD with 58%, but also a decrease in liver enzymes by MF in hyperinsulinemic, overweight patients with PCOS could be demonstrated [44]. Preiss et al. found a decrease of ALT levels in obese women with PCOS treated with MF which was associated with body weight improvement [43]. This is in line with some small studies in patients with biopsy proven NAFLD and/or NASH, which also showed improvement of liver function under MF therapy [33–37]. Our study shows that, in insulin resistant patients, the positive effect of MF on liver function seems to be limited to those who are able to achieve insulin sensitivity. Furthermore, improvement of LIFL by MF can also be demonstrated in patients who are insulin sensitive.

Concerning the influence of MF on NASH, studies including patients with biopsy proven NAFLD and/or NASH show divergent results in regard to the effect of MF on histological indices. An Italian study examining the effect of MF versus vitamin E and versus diet in nondiabetic NAFLD showed a decrease of liver fat, necroinflammation, and fibrosis in some patients who were treated with MF and underwent liver rebiopsy. However, in this study, no biopsy was undertaken in patients of the other therapy regimes due to concerns of the local ethical committee [35]. In a Turkish study, patients with biopsy proven NASH were treated with therapeutic lifestyle changes alone or in combination with insulin sensitizers (MF or rosiglitazone). Treatment with insulin sensitizers led to significant improvement of NASH activity score, while grade of fibrosis did not change [37]. In a small, long-term MF treatment study of NAFLD patients over one year, Nair et al. observed only a transient improvement of liver enzymes and a modest beneficial effect with improvement of histologically assessed steatosis in 33% and of inflammation and fibrosis in 20% and 10% of cases, respectively [40]. Uygun et al. could not demonstrate a significant effect on histopathological findings of liver rebiopsy in MF/diet-treated NASH-patients, although patients in the MF regime showed significantly greater weight loss and improvement of IR and liver function than the patients undergoing diet alone [34]. Two placebo controlled intervention studies in NAFLD or NASH-patients, respectively, one with higher and one with lower MF than usually used (3000 mg and 500 mg/d), did not show a positive effect of MF on liver histology [41, 42]. Unchanging levels of M30 and unchanged prevalence of sNASH in our study possibly support the missing effect of MF seen in these studies with histological NAFLD assessment, though, considering that patients with persistent IR exhibited an increase of liver enzymes and apoptotic markers, the unchanged apoptosis marker in association with lower liver enzymes could also be interpreted as stabilization of hepatic damage. In contrast, patients who fail to normalize IR might be at greater risk for progression of NAFLD as suggested by increasing serum apoptosis markers and liver enzymes. However, the mentioned changes in M30 levels in the PCOS-PIR group did not reach statistical significance and patient number with sNASH in our groups is low. Another explanation might be a longer duration needed for NAFLD or NASH to get cleared. Taken together, IR alone might not be accountable for development of NAFLD and the impact of MF on NAFLD seems limited or might require a longer observation time to be fully understood.

Another finding of this study was that patients with PCOS who failed to achieve normalization of HOMA-IR were significantly more obese than the other two groups. Loomba and colleagues reported in their subanalysis of histological responders versus nonresponders that histological response under MF treatment in NASH patients was associated with loss of body weight of at least 5 kg and that none of the patients with a BMI > 40 kg/m² at baseline reached histological response to MF therapy [38]. Patients with PCOS who are severely obese and/or insulin resistant without response to MF should possibly be monitored more closely as they are potentially at higher risk for progression of NAFLD from simple steatosis towards NASH.

The presented study has several limitations, which need to be taken into account. The examined study population is quite small and we performed this analysis as an observational study. Patients with PCOS without IR represented a control population assuming that in these patients MF will not influence hepatic markers. Though no randomized trial with a placebo group was performed, we cannot exclude that observed results are a time effect. Despite these limitations, this study shows preliminary data about the effect of MF on hepatic injury and apoptosis in a cohort of patients with PCOS.

In summary, we found a significant improvement of liver enzymes in subgroups of patients with PCOS without change in hepatic apoptotic markers. Whether the latter means a missing effect of MF on NAFLD or can be interpreted as prevention of progression is unclear. Further studies are needed to elucidate NAFLD in the context of PCOS and its potential therapeutic options. Studies evaluating the therapeutic effect of insulin sensitizing agents like MF on NAFLD should possibly take the patients' response regarding insulin resistance into account.

## Figures and Tables

**Table 1:** Baseline characteristics of patients with PCOS. Comparison of baseline characteristics of PCOS patients with (PCOS-IR) and without IR (PCOS-C). Data were analyzed by *t*-tests, Mann-Whitney *U* test (for BARD-Scores), or Chi²-tests (for dichotomous variables). Values are given as mean ± standard deviation, unless otherwise indicated.

Parameter	PCOS-C (*n* = 36)	PCOS-IR (*n* = 53)	*P*
HOMA-IR [mmol∗mU/L²]	1.4 ± 0.7	4.9 ± 2.6	0.0001
Fasting insulin [*µ*U/mL]	6.3 ± 3.1	20.8 ± 9.9	0.0001
AUCI [mU∗h/L]	127 ± 48	300 ± 141	0.0001
Testosterone [nmol/L]	2.5 ± 0.9	2.7 ± 0.8	NS
FAI	4.7 ± 3.3	13.2 ± 16.6	0.001
BMI [kg/m²]	26.8 ± 6.4	35.8 ± 8.5	0.0001
Body weight [kg]	75.9 ± 21.1	101.0 ± 25.0	0.0001
Cholesterol [mg/dL]	186 ± 28	188 ± 33	NS
LDL-cholesterol [mg/dL]	102 ± 22	112 ± 30	NS
HDL-cholesterol [mg/dL]	63 ± 14	48 ± 10	0.0001
Triglycerides [mg/dL]	84 ± 48	161 ± 166	0.002
MBS [%]	5.6	69.8	0.0001
AST [U/L]	25 ± 29	21 ± 6	NS
ALT [U/L]	26 ± 14	30 ± 12	NS
AST/ALT-ratio	0.89 ± 0.3	0.72 ± 0.2	0.002
LIFL [%]	22.2	35.8	NS
BARD-Score [median (25th, 75th percentile)]	2 (0.25, 2.75)	1 (1, 3)	NS
M30 [U/L]	198 ± 91	220 ± 170	NS
NASH [%]	5.6	5.7	NS

**Table 2:** Outcome parameters at baseline and after 6-month metformin treatment for patients with PCOS who were insulin sensitive at baseline (PCOS-C), patients with IR at baseline and after treatment (PCOS-PIR),  and initially insulin resistant patients whose IR dissolved after treatment (PCOS-exIR).

	PCOS-C^*^		PCOS-exIR^*^		PCOS-PIR^*^		
	*n* = 36		*n* = 25		*n* = 28		Two-factorial ANOVA or Wilcoxon test^a^
	Baseline	After treatment	Baseline	After treatment	Baseline	After treatment	
HOMA-IR >2.5 [%]	0	5.6	100	0	100	100	

HOMA-IR [mmol∗mU/L²]	1.4 ± 0.7	1.1 ± 0.8	3.6 ± 0.9	1.5 ± 0.7^***^	6.2 ± 3.0	4.4 ± 1.8^**^	**Time ** **F** = 44.5, **P** < 0.001 **Group ** **F** = 88.3, **P** < 0.001 **Time × Gr ** **F** = 8.5, **P** < 0.001

Fasting insulin [*µ*U/mL]	6.3 ± 3.1	5.3 ± 3.3	15.0 ± 3.4	7.0 ± 2.9^***^	25.9 ± 10.9	19.7 ± 7.5^**^	**Time ** **F** = 50.2, **P** < 0.001 **Group ** **F** = 98.8, **P** < 0.001 **Time × Gr ** **F** = 9.0, **P** < 0.001

AUCI [mU∗h/L]	127 ± 48	132 ± 55	247 ± 109	169 ± 76^***^	347 ± 151	356 ± 142	**Time ** **F** = 4.1, **P** = 0.046 **Group ** **F** = 49.7, **P** < 0.001 **Time × Gr ** **F** = 6.6, **P** = 0.002

BMI [kg/m²]	26.8 ± 6.4	26.6 ± 6.4	31.9 ± 6.9	29.8 ± 6.3^***^	39.2 ± 8.4	38.5 ± 8.4	**Time ** **F** = 20.5, **P** < 0.001 **Group ** **F** = 23.8, **P** < 0.001 **Time × Gr ** **F** = 6.3, **P** < 0.003

Body weight [kg]	75.9 ± 21.1	75.2 ± 21.3	90.2 ± 21.7	84.4 ± 19.4^***^	110.6 ± 24.2	108.9 ± 24.0	**Time ** **F** = 19.6, **P** < 0.001 **Group ** **F** = 19.6, **P** < 0.001 **Time × Gr ** **F** = 6.3, **P** = 0.003

Cholesterol [mg/dL]	186 ± 28	196 ± 34	182 ± 32	186 ± 39	192 ± 34	190 ± 26	Time *F* = 0.9, *P* = 0.33 Group *F* = 0.5, *P* = 0.59 Time × Gr *F* = 1.1, *P* = 0.35

LDL-cholesterol [mg/dL]	102 ± 22	105 ± 28	106 ± 26	103 ± 31	117 ± 32	113 ± 22	Time *F* = 0.1, *P* = 0.82 Group *F* = 2.1, *P* = 0.13 Time × Gr *F* = 0.5, *P* = 0.61

HDL-cholesterol [mg/dL]	63 ± 14	63 ± 15	49 ± 11	53 ± 13	48 ± 10	50 ± 11	**Time ** **F** = 4.5, **P** = 0.037 **Group ** **F** = 13.8, **P** < 0.001 Time × Gr *F* = 1.2, *P* = 0.33

Triglycerides [mg/dL]	84 ± 48	101 ± 43	128 ± 70	122 ± 51	191 ± 217	153 ± 68	Time *F* = 0.6, *P* = 0.45 **Group ** **F** = 7.4, **P** = 0.001 Time × Gr *F* = 1.8, *P* = 0.17

AST [U/L]	25 ± 29	17 ± 5	20 ± 5	18 ± 4^(∗)^	21 ± 6	21 ± 8	Time *F* = 2.3, *P* = 0.13 Group *F* = 0.26, *P* = 0.78 Time × Gr *F* = 1.6, *P* = 0.22

ALT [U/L]	26 ± 14	21 ± 6^(∗)^	28 ± 10	23 ± 9^**^	32 ± 13	38 ± 18^(∗)^	Time *F* = 0.92, *P* = 0.34 **Group ** **F** = 10.6, **P** < 0.001 **Time × Gr ** **F** = 7.6, **P** < 0.001

AST/ALT-ratio	0.89 ± 0.3	0.86 ± 0.2	0.76 ± 0.2	0.86 ± 0.2	0.69 ± 0.2	0.61 ± 0.2	Time *F* = 0.33, *P* = 0.56 **Group ** **F** = 11.7, **P** < 0.001 Time × Gr *F* = 2.3, *P* = 0.06

M30 [U/L]	198 ± 91	206 ± 124	199 ± 108	196 ± 70	238 ± 211	275 ± 206	Time *F* = 1.8, *P* = 0.19 Group *F* = 1.7, *P* = 0.19 Time × Gr *F* = 1.2, *P* = 0.31

BARD-Score [median (25th, 75th percentile)]	2 (0.25, 2.75)	2 (1, 2.75)	1 (1, 3)	2 (1, 3)	1 (1, 2.5)	1 (1, 1)	Time *Z* = 0.3, *P* = 0.79

^a^Data were analyzed with repeated measures analysis of variance with the factors group (i.e., PCOS-C,  PCOS-IR, and PCOS-exIR) and time (i.e.,  changes from baseline to 6 months of treatment). In case of significant ANOVA time or time × group effects, post hoc paired *t*-tests within respective PCOS groups were calculated (^*^ *P* < 0.05, ^**^ *P* < 0.01, ^***^ *P* < 0.001, and ^(∗)^nonsignificant after Bonferroni correction). Changes in BARD-Scores from baseline to 6 months after treatment were analyzed with Wilcoxon tests within subgroups and the total sample. All results are shown as mean ± standard deviation,  unless otherwise indicated.

## Abbreviations

PCOS: Polycystic ovary syndrome
IR: Insulin resistance
NAFLD: Nonalcoholic fatty liver disease
SH: Steatosis hepatis
NASH: Nonalcoholic steatohepatitis
CK18: Cytokeratin 18
AST: Aspartate aminotransferase
MF: Metformin
FAI: Free androgen index
MS: Metabolic syndrome
ALT: Alanine aminotransferase
PCOS-IR: PCOS patients with IR
PCOS-C: PCOS patients without IR
PCOS-PIR: PCOS with persistent IR
PCOS-exIR: PCOS with dissolved IR.
